# Crippling of *Klebsiella pneumoniae* virulence by metformin, N-acetylcysteine and secnidazole

**DOI:** 10.1186/s12866-023-02969-9

**Published:** 2023-08-22

**Authors:** Shokri M. Shafik, Hisham A. Abbas, Nehal Yousef, Moustafa M. Saleh

**Affiliations:** 1 Microbiology and Immunology Department, Faculty of Pharmacy, Zagazig University, Zagazig City, Egypt; 2 Microbiology and Immunology Department, Faculty of Pharmacy, Port Said University, Port Said City, Egypt

**Keywords:** *Antibiotic resistance*, *K. pneumoniae*, *Metformin*, *N-acetylcysteine*, *Secnidazole*, *Virulence inhibitors*

## Abstract

**Introduction:**

The emergence of multidrug-resistant Klebsiella pneumoniae in hospitals represents a serious threat to public health. Infections caused by Klebsiella pneumoniae are widespread in healthcare institutions, mainly pneumonia, bloodstream infections, and infections affecting neonates in intensive care units; so, it is necessary to combat this pathogen with new strategies. Targeting virulence factors necessary to induce host damage and disease is a new paradigm for antimicrobial therapy with several potential benefits that could lead to decreased resistance.

**Background:**

The influence of metformin, N-acetylcysteine, and secnidazole on Klebsiella pneumoniae virulence factors production was tested. The production of Klebsiella pneumoniae virulence factors such as biofilm formation, urease, proteases, hemolysins, and tolerance to oxidative stress was evaluated phenotypically using sub-inhibitory concentration (1/8 MIC) of metformin, N-acetylcysteine, and secnidazole. For more confirmation, qRT-PCR was used to assess the relative expression level of rmpA, wcaG, fimH-1, mrkD, ureA, and khe genes regulating virulence factors production.

**Results:**

Metformin, N-acetylcysteine, and secnidazole were all found to have a powerful inhibitory effect on the production of virulence factors phenotypically. Our results showed a significant reduction in the expression level of rmpA, wcaG, fimH-1, mrkD, ureA, and khe genes. Furthermore, the tested drugs were investigated in vivo to inform their ability to protect mice against Klebsiella pneumoniae pathogenesis.

**Conclusions:**

Metformin, N-acetylcysteine, and secnidazole inhibited the virulence of Klebsiella pneumoniae. Besides combating resistant Klebsiella pneumoniae, the tested drugs could also serve as an adjuvant to traditional antibiotics.

## Introduction

Humans are colonized by *Klebsiella pneumoniae* (*K. pneumoniae*), an encapsulated, Gram-negative non-motile bacterial pathogen. It is a member of the normal gastrointestinal tract and nasal flora and causes no diseases in healthy individuals, but it is an opportunistic pathogen that causes several types of infections in immunocompromised individuals [1, 2].* K. pneumoniae* may cause meningitis, bloodstream infections, and surgical site infections [3].

*K. pneumoniae* was reported from around the world causing outbreaks of neonatal infections, particularly in premature infants [4]. In recent years, multiple studies have documented outbreaks in hospitals caused by multidrug-resistant (MDR) *K. pneumoniae* isolates [5]. Even in developed countries, neonatal sepsis caused by MDR *K. pneumoniae* is a common occurrence in neonatal intensive care units (NICUs) and has significant morbidity and mortality rates [6]. Developing novel approaches to combat MDR *K. pneumoniae* infections is a major health and economic challenge [7].

The pathogenicity of* K. pneumoniae* is attributed to the production of many virulence factors including, capsular polysaccharide, biofilm formation, urease, proteases, and hemolysins production [8].* K. pneumoniae* virulence factors are encoded by certain virulence-regulating genes including *rmpA, wcaG, fimH-1, mrkD, ureA,* and *khe* [9, 10].

Several advantages over traditional antibiotics can be found in targeting virulence factors. This includes a broader repertoire of pharmacological targets, the development of novel antimicrobials, the reduction of resistance, and the preservation of normal microbiota [11, 12]. The World Health Organization (WHO) has warned that the world is on the verge of entering a post-antibiotic era [13]. It's noteworthy that repurposing drugs as a new anti-virulence therapy is a very promising strategy [14]. Many previous studies have reported the repurposing of many FDA-approved drugs as promising anti-virulence agents [15].

Metformin is the most widely used type II diabetes oral hypoglycemic drug, with potential applications in other pathological conditions such as weight management, cardio-protection, and neuro-protection [16]. According to previous research findings, metformin is proven to be used as a newer efflux pump inhibitor in *K. pneumoniae* [17], and as a quorum sensing (QS) inhibitor in *P. aeruginosa* [18].

In addition, N-acetylcysteine (NAC) is widely used as the specific antidote for acetaminophen overdose and has the potential to be used as an adjuvant in the treatment of a variety of medical conditions [19, 20]. In previous reports, NAC was found to have a significant impact on inhibiting the adherence of *S. aureus*, *K. pneumoniae*, *P. aeruginosa,* and *Proteus mirabilis* on the surface of catheters, and decreasing their biofilm formation activity [21]. Moreover, the use of NAC with β-lactams was reported to augment the antibiotic activity against *P. aeruginosa* [22].

Another FDA-approved drug, secnidazole, which is used as an anti-protozoal, has been reported in recent investigations to have a strong inhibitory effect on *P. aeruginosa* virulence factors production [13], and was reported to have a promising anti-virulence activity that could be employed to combat *Serratia marcescens* infections [23].

In the present study, we aimed to investigate the potential inhibitory effect of metformin, NAC, and secnidazole on the production of *K. pneumoniae* virulence factors. Our results could improve trials trying to find an alternative solution to combat *K. pneumoniae* infections and reduce the incidence of antibiotic resistance in this serious pathogen.

## Methods

### Media and chemicals

Mueller Hinton agar (MHA), Tryptone soy broth (TSB), and Luria–Bertani (LB) broth were purchased from Oxoid, Hampshire, England. Metformin was obtained from Sigma Pharmaceutical Industries, Egypt. EIPICO pharmaceutical company provided the secnidazole, while NAC was the product of SEDICO pharmaceutical company, Egypt. Other chemicals were of pharmaceutical grade.

### Animals

Three-week-old healthy albino mice (*Mus musculus*) were purchased from the animal house of the Animal House of the Faculty of Veterinary Medicine, Mansoura University, Egypt, and kept under observation for at least one week prior to study with free access to food and water. The ethical standards for animal welfare approved by The Institutional Animal Care and Use Committee, Zagazig University (ZU-IACUC), Egypt approved the study (No: ZU-IACUC/3/F/141/2022). All procedures were performed according to ARRIVE guidelines. At the end of the study, all animals were euthanized by thiopental (intravenous injection, 150 mg/kg).

### Bacterial isolates and their identification

Ten clinical isolates of *K. pneumoniae* were used in the current study. There were obtained from the stock culture collection of Microbiology and Immunology Department, Faculty of Pharmacy, Zagazig University. These isolates were recovered previously from neonates admitted to Nabrouh Central Hospital Intensive Care Unit, Egypt who had sepsis.

The identity of the isolates was fully confirmed by using VITEK® 2 COMPACT with VITEK® ID GNB card (Biomerieux, France) according to the manufacturer’s instructions [24].

### Antibiotic susceptibility testing of the tested clinical isolates

The antibiotic susceptibility testing for the clinical isolate was carried out by minimum inhibitory concentration (MIC) determination using the VITEK 2 COMPACT automated machine. AST-N20 card was used to perform antimicrobial susceptibility tests for gram-negative bacteria against sixteen antibiotics namely, ampicillin, ampicillin/sulbactam, cefazolin, ceftriaxone, cefepime, aztreonam, imipenem, meropenem, amikacin, gentamicin, tobramycin, ciprofloxacin, moxifloxacin, tigecycline, nitrofurantoin, Trimethoprim/sulfamethoxazole according to the manufacturer label.

### Determination of the MIC of metformin, NAC, and secnidazole

The broth microdilution technique was used to determine the MIC of metformin, NAC, and secnidazole according to Clinical and Laboratory Standards Institute (CLSI) [25]. Briefly, a colony of bacteria was incubated overnight in MHB, then diluted in fresh MHB to obtain a cell density of 10^6^ CFU/ml. After preparing twofold serial dilutions of the examined chemical substances in MHB, 100 μL of the diluted potential inhibitor solutions were inoculated with 100 μL of microbial suspensions in microtiter plates. We calculated the MIC values of metformin, NAC, and secnidazole by measuring the lowest concentration of the tested substances that inhibited the visible growth of microorganisms after incubation for 24 h at 37 °C.

### Evaluation of the effect of sub-MICs of Metformin, NAC, and secnidazole on the growth kinetics of *K. pneumoniae*

To evaluate the impact of sub-minimum inhibitory concentrations (sub-MICs) of the test drugs on *K. pneumoniae* growth, a growth curve experiment was performed. Briefly, the isolates were cultured in LB until an OD600 of 0.2 was reached. The culture was then aliquoted into four 500 mL Erlenmeyer flasks. 1/8 MIC of Metformin, NAC, and secnidazole were added to three of the flasks, while the fourth flask served as an untreated control. The flasks were incubated at 37 °C with shaking. The OD600 was measured every 30 min. Samples of 1 mL were taken immediately after addition of the inhibitors (t_0_), and then at 30, 60, 90, 120, 150, 180, 210, 240, 270, 300, 330 and 360 min. This was done to determine the effect of sub-MICs of the drugs on *K. pneumoniae* growth over time. The data are the average of the three independent experiments [26].

### Phenotypic assay of inhibition of virulence factors

#### Biofilm inhibition assay

In brief, a suspension of the tested isolates in TSB was prepared from overnight culture and adjusted to 10^6^ CFU/ml. Aliquots of 200 µl of the bacterial suspension were transferred to the wells of a microtiter plate in the presence and absence of sub-MIC of the tested drugs and incubated at 37 °C for 48 h. The TSB was gently removed, and the plates were washed with distilled sterile water to eliminate any planktonic cells, followed by air drying. After 20 min of treatment with 200 µl of 99% fixing methanol, the biofilm was stained for 15 min with 200 µl of 1% crystal violet solution. After washing the plate, crystal violet was dissolved in 33% glacial acetic acid, and the absorbance of the solubilized dye was measured at 570 nm using a Biotek Spectrofluorometer. The test was performed in triplicate, and the percentage of inhibition was calculated [27].

#### Urease inhibition assay

Bacterial urease production was determined by a modified colorimetric assay [8]. Briefly, the bacteria were grown overnight in urea broth (0.1 g/L yeast extract, 9.1 g/L KH2PO4, 9.5 g/L Na2HPO4, 20 g/L urea, phenol red 0.01 g/L) containing sub-MICs of the tested drugs. Drug-free urea broth tubes were used as a control. After incubation for 24 h, the suspensions were centrifuged for three minutes at 5000 g. The supernatant was used to determine the activity of the urease. Color changes from yellow to red, and color intensity was measured using a Biotek spectrophotometer at 560 nm. The test was performed in triplicate, and the percentage of inhibition was calculated.

#### Total protease activity inhibition assay

The total proteases produced in the presence and absence of the tested drugs were estimated by the modified skim milk assay [28]. After overnight growth of *K. pneumoniae* in LB broth with and without 1/8 MIC of the tested agents, supernatants were centrifuged at 10,000 g for 10 min. To measure the proteolytic activity of each isolate, 1 ml of cell-free supernatant from each isolate was mixed with 1 ml skim milk solution (1.25% in distilled sterile water) and incubated for 30 min at 37 °C. As a measure of proteolytic activity, the turbidities of assay solutions were assessed at OD_600_ using a Biotek spectrofluorometer. The test was performed in triplicate, and the percentage of inhibition was calculated.

#### Hemolysin activity inhibition assay

The hemolysins assay was evaluated by mixing 600 μl of cell-free supernatants prepared as mentioned in the protease assay with and without 1/8MIC of the tested drugs with 600 μl of fresh 2% v/v defibrinated rabbit blood cells in saline. It was centrifuged at 10,000 g for 8 min at 4 °C after being incubated at 37 °C for 2 h. Using a Biotek spectrofluorometer, released hemoglobin was measured at 540 nm and compared with both negative and positive controls (erythrocytes incubated in LB broth and erythrocytes lysed completely with 0.1% SDS, respectively). To calculate hemolysis percentage %, we used the formula [X-B/T-B] × 100, where X stands for the drug-treated or untreated isolates, B stands for the negative control, and T represents a positive control. The test was performed in triplicate, and the percentage of inhibition was calculated [26].

### Testing sensitivity to oxidative stress

To investigate the interfering capacity of metformin, NAC, and secnidazole with resistance to oxidative stress in *K. pneumoniae*, the modified disk test method was used [29]. Isolates were cultured in TSB in the presence or absence of the tested drugs and incubated overnight. On top of TSA plates containing 1/8 MIC of the tested drugs, 0.1 ml of bacterial suspension was added to the surface of TSA plates and spread with a glass spreader. The TSA plates were then coated with sterile paper discs and hydrogen peroxide (1.5%) was added. After incubating the plates for 24 h at 37 °C, the inhibition zones were measured. The test was performed in triplicate, and the percentage of inhibition was calculated.

### RNA extraction and relative gene expression assessment using qRT-PCR

The most resistant isolate (isolate 1) was selected as a representative isolate for estimating the relative expression of genes associated with the production of virulence factors in *K. pneumoniae*. The isolate was cultured overnight at 37 °C in TSB in the presence and absence of sub-MIC of metformin, NAC, and secnidazole until the bacteria reach the middle log phase (OD_600_ 0.5–0.6). The pellets were obtained by centrifugation at 6000 g for 15 min and RNA was extracted using GeneJET RNA Purification Kit (Thermo Fisher Scientific Inc., Germany) according to manufacture instructions. Reverse transcription followed by qRT-PCR of virulence factors genes *khe, fimH-1, mrkD, ureA, rmpA and wcaG* was performed following the protocol described in SensiFAST™ SYBR® Hi-ROX One-Step Kit (Bioline, UK). The qRT-PCR analysis was set up via StepOne RT-PCR thermal cycler (Applied Biosystem, USA) using primers described in Table 1. The housekeeping gene 16S rRNA was used to standardize the relative expression values of each gene. The 2^−∆∆CT^ approach was used to compare the relative gene expression in the treated and untreated isolates [30].

**Table 1:** Primers for virulence genes used in qRT-PCR

Gene name	Primer sequence (5′ → 3′)	Reference
*khe*	F: GATGAAACGACCTGATTGCATTC	[31]
	R: CCGGGCTGTCGGGATAAG	
*fimH-1*	F: GCC AAC GTC TAC GTT AAC CTG	[32]
	R: ATA TTT CAC GGT GCC TGA AAA	
*mrkD*	F: AAGCTATCGCTGTACTTCCGGCA	[33]
	R: GCGTTGGCGCTCAGATAGG	
*ureA*	F: GACAAGCTGTTGCTGTTTACC	[34]
	R: CGGGTTGTGAACGGTGAC	
*rmpA*	F; ACTGGGCTACCTCTGCTTCA	[35]
	R: CTTGCATGAGCCATCTTTCA	
*wcaG*	F: GGTTGGKTCAGCAATCGTA	[32]
	R: ACTATTCCGCCAACTTTTGC	
16 s rRNA	F: GCAAGTCGAGCGGTAGCACAG	[36]
	R: CAGTGTGGCTGGTCATCCTCTC	

a = Forward, b = Reverse

#### Mice survival assay

The protective activities of metformin, NAC, and secnidazole against *K. pneumoniae* pathogenesis were investigated in the mice survival model [37]. The *K. pneumoniae* cultures with or without sub-MIC of the tested drugs were adjusted to 1 × 10^8^ CFU/ml in phosphate-buffered saline (PBS). Six groups of albino mice (*Mus musculus*) of similar weight and health characteristics were studied, each group containing 10 mice. The two negative control groups were administered 100 μl of sterile PBS intraperitoneally in one group, and the other was left untreated and un-inoculated. As a positive control, 100 μl of untreated *K. pneumoniae* was injected into a group of subjects. Each of the other three groups received 100 μl of overnight cultures in LB broth containing sub-MICs of metformin, NAC, and secnidazole, respectively. A survival rate was determined by observing experimental mice for three consecutive days.

### Statistical analysis

The GraphPad Prism 8.0.2 software package was used to analyze the data. The effect of the tested drugs on *K. pneumoniae* virulence factors was compared using One Way ANOVA at *P* > 0.05 and > 0.001 for significance. Calculations were based on means and standard errors of three biological experiments conducted with three technical replicates.

## Results

### Clinical isolates identification and antibiotic susceptibility

The clinical isolates in the current study were confirmed as *K. pneumoniae* using VITEK 2 COMPACT system (BIOMERIEUX) for bacterial bio-typing and antibiotic susceptibility patterns.

A high proportion of the tested isolates were resistant to the antibiotics tested. Of the 10 isolates, 100% resistance was found against each of ampicillin, and aztreonam. On the other hand, the resistance rates were 90% against ampicillin/sulbactam, imipenem, and ceftriaxone (9 isolates out of 10), 80% against cefazolin, meropenem, gentamicin, and nitrofurantoin (8 isolates), 70% against cefepime (7 isolates out of 10), 60% against ciprofloxacin (6 isolates out of 10), and 40% against sulfamethoxazole/trimethoprim (4 isolates out of 10). Tigecycline was the most effective antibiotic with a resistance rate of 10% (one isolate out of 10). The complete results of antibiotic susceptibility testing are illustrated in Table 2. Data from antibiotic sensitivity tests revealed that 20% of the isolates (2 out of 10) were XDR. 80% of the isolates were MDR (8 out of 10).

**Table 2:** Antibiotic susceptibility pattern of the tested clinical isolates

Isolates Antibiotics	Isolate 1	Isolate 2	Isolate 3	Isolate 4	Isolate 5	Isolate 6	Isolate 7	Isolate 8	Isolate 9	Isolate 10
Ampicillin	R	R	R	R	R	R	R	R	R	R
Ampicillin /Sulbactam	R	R	R	R	S	R	R	R	R	R
Cefazolin	R	R	S	R	S	R	R	R	R	R
Ceftriaxone	R	R	S	R	R	R	R	R	R	R
Cefepime	R	R	S	I	R	R	R	S	R	R
Aztreonam	R	R	R	R	R	R	R	R	R	R
Imipenem	R	R	R	R	R	R	R	S	R	R
Meropenem	R	R	R	R	R	R	S	S	R	R
Amikacin	R	R	S	S	I	S	S	S	I	R
Gentamicin	R	R	R	R	R	R	R	S	R	I
Tobramycin	R	R	R	S	R	I	I	I	R	R
Ciprofloxacin	R	R	I	S	R	S	S	R	R	R
Moxifloxacin	R	I	I	S	S	S	S	R	S	R
Tigecycline	R	S	S	S	S	S	S	S	S	S
Nitrofurantoin	R	R	R	R	R	I	R	I	R	R
Trimethoprim/ sulfamethoxazole	R	S	R	R	S	S	S	S	S	R

*R* Resistant, *S* Susceptible, *I* Intermediate

### Determination of the MIC of metformin, NAC, and secnidazole

Metformin, NAC, and secnidazole inhibited the growth of the tested *K. pneumoniae* isolate at 8 mg/ml, 16 mg/ml, and 8 mg/ml, respectively. The inhibitory activities against *K. pneumoniae* virulence factors were tested at concentrations equivalent to 1/8 MIC of the tested drugs, (1 mg/ml) for metformin, (2 mg/ml) for NAC, and (1 mg/ml) for secnidazole as other sub-inhibitory concentrations (0.5 and 0.25 mg/ml) did not show any inhibitory impact on virulence factors production by phenotypic tests.

### Evaluation of the non-lethality of metformin, NAC, and secnidazole at sub-MICs on *K. pneumoniae* growth

**Fig. 1:**
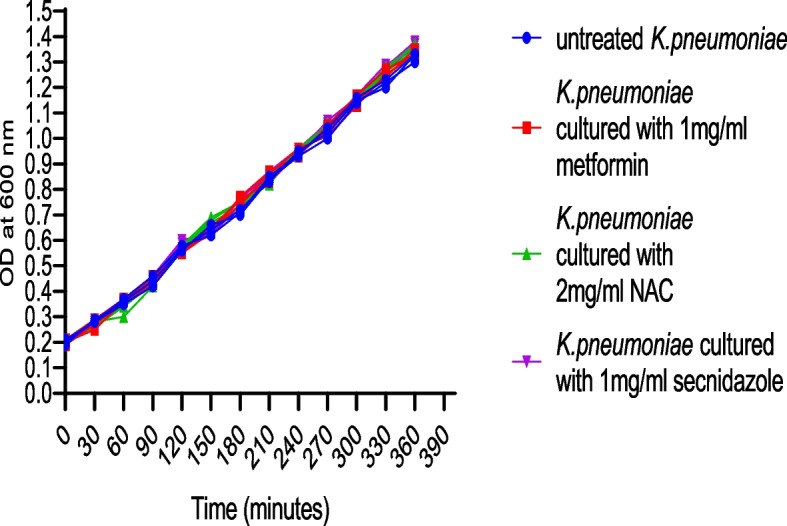
Effect of sub-MICs of metformin, NAC, and secnidazole on the growth of *K. pneumoniae*. *K. pneumoniae* was cultured in LB broth and the OD600 was measured every 30 min with or without the addition of 1/8 MIC of metformin, NAC, and secnidazole. The growth curves were compared to that of the untreated bacteria cultured in LB broth only. The results show that sub-MICs of metformin, NAC, and secnidazole did not significantly alter the growth of *K. pneumoniae*. The test was performed in triplicate. Values are the averages of three independent experiments

A growth curve assay was performed to evaluate the effect of sub-minimum inhibitory concentrations (sub-MICs) of the test drugs on *K. pneumoniae* growth kinetics. The optical density at 600 nm (OD600) was measured every 30 min to generate growth curves for each condition. Figure 1 shows that at 1/8MIC, none of the test drugs caused a significant change in the growth curve of *K. pneumoniae* compared to the untreated control. This confirms that the test drugs did not exhibit lethal effects on *K. pneumoniae* at the sub-MICs used in this study.

### The effect of metformin, NAC, and secnidazole on the production of *K. pneumoniae* virulence factors

#### Biofilm inhibition assay

**Fig. 2:**
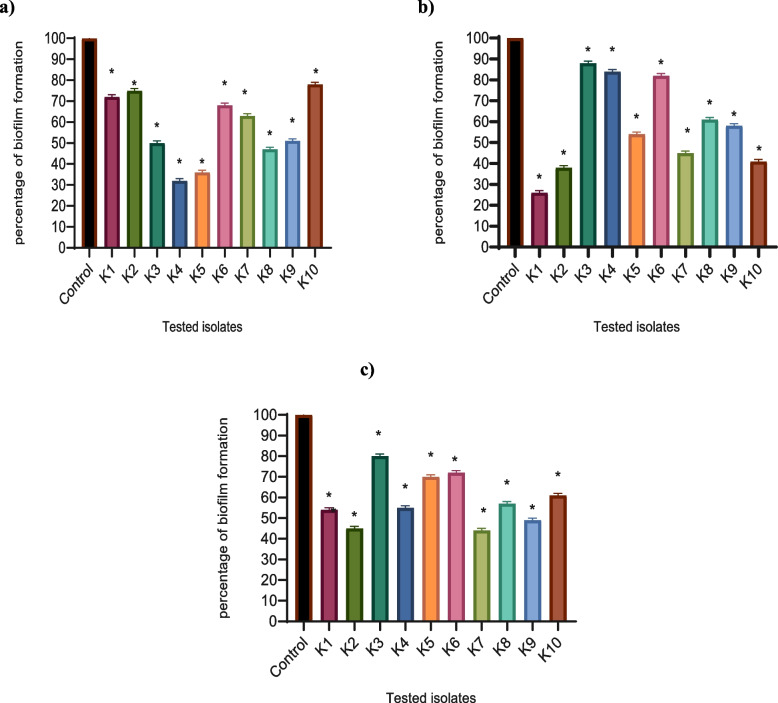
Inhibition of biofilm formation in *K. pneumoniae* by **a**) metformin, **b**) NAC, and **c**) secnidazole. A significant reduction of biofilm formation was found with 1/8 MIC of metformin, NAC, and secnidazole in the treated isolate in comparison with the untreated isolate. Biofilm formation was measured by staining with crystal violet and the OD570 was measured. The results were expressed as percentage of OD. The data shown represents the means ± standard errors. One WAY ANOVA test was used for statistical analysis. *, significant *P* < 0.05 was considered significant

The inhibition of biofilm formation was evaluated using the crystal violet method. Interestingly, metformin, NAC, and secnidazole significantly reduced the biofilm formation ability of *K. pneumoniae*. The percentages of biofilm formation were reduced by a percentage ranging between (22%-68%), (12%-67%), and (20%-56%) in metformin, NAC, and secnidazole-treated isolate, respectively in comparison to the control untreated isolates (Fig. 2).

#### Urease activity production assay

**Fig. 3:**
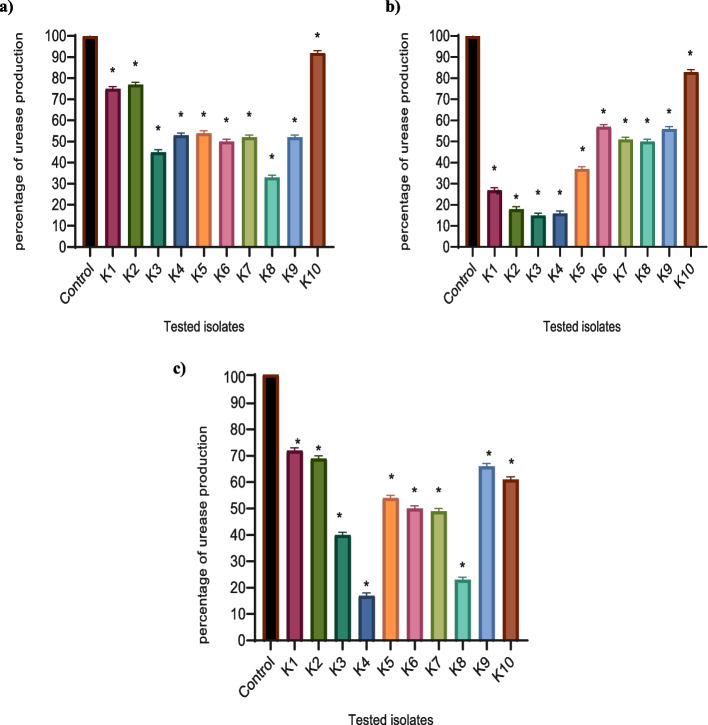
Urease production was significantly reduced in **a**) metformin, **b**) NAC, and **c**) secnidazole-treated isolates as compared to control untreated bacteria. The absorbance of urease was measured at 560 nm after overnight incubation in urea broth. The OD values of the treated isolates were expressed as a percentage of the OD values compared with untreated control isolates. The results shown are the means ± standard errors of three biological experiments with three technical replicates each. *, significant *P* < 0.05 (following One Way ANOVA) was considered significant

Metformin, NAC, and secnidazole-treated isolates showed a remarkable reduction in urease production (Fig. 3). The production in treated isolates was reduced by a percentage ranging between (8%-67%) by metformin, (17%-85%) by NAC, and (21%-83%) by secnidazole, respectively in comparison to control untreated isolates.

#### Total protease activity inhibition assay

**Fig. 4:**
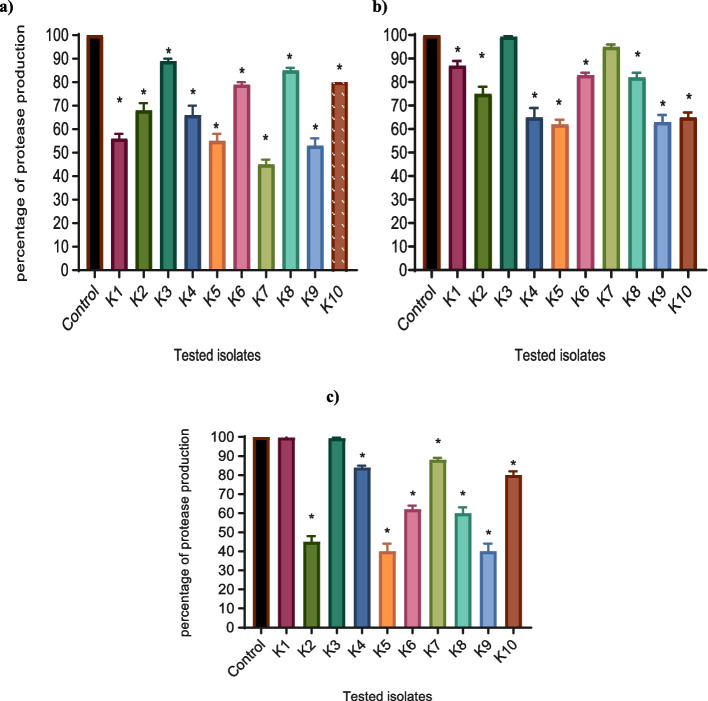
Significant reduction of protease production was found with **a**) metformin, **b**) NAC, and **c**) secnidazole-treated isolates. OD_600_ was measured following overnight culturing of bacteria in LB broth in the presence and absence of 1/8 MIC of tested isolates followed by incubation of supernatants with skim milk for 30 min at 37 °C. The OD values of the treated strains were expressed as a percentage of the OD values compared with the untreated control strain. The data shown are the means ± standard errors of three biological experiments with three technical replicates each. *, significant *P* < 0.05

The proteolytic activity was screened using the modified skim milk assay method in the presence and absence of sub-MICs of the tested drugs. The highest significant inhibitory effect of protease activity was obtained in a percentage ranging between (20%-55%) by metformin. NAC and secnidazole also showed a significant decrease in protease activity by a percentage ranging between (17%-38%), and (16%-60%) respectively (Fig. 4).

#### Hemolysin activity inhibition assay

**Fig. 5:**
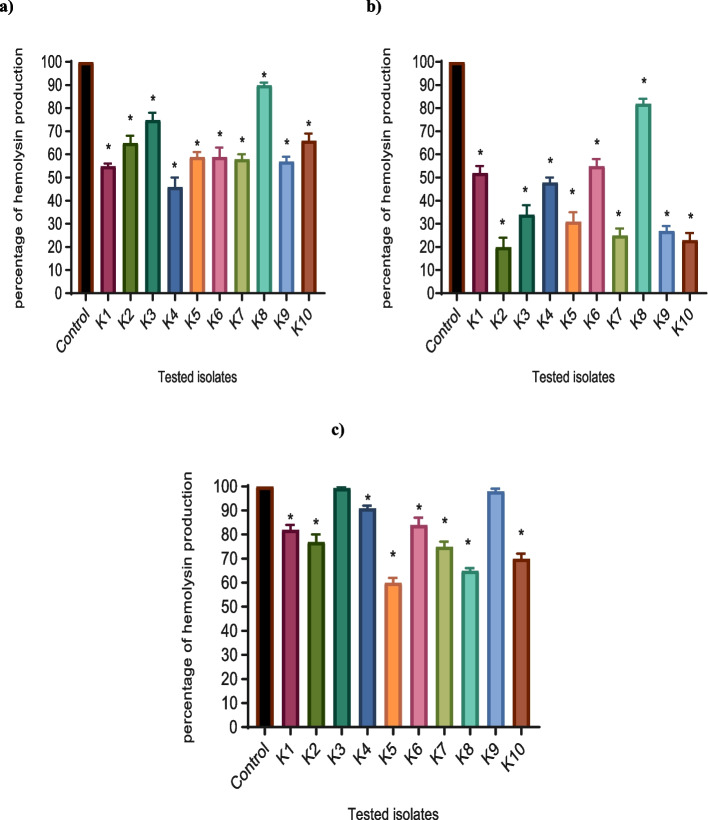
A significant decrease in hemolytic activity was found with **a**) metformin, **b**) NAC, and **c**) secnidazole-treated isolates. The hemolytic activity of the drug-free supernatant was considered as 100% hemolysis (control), and the percentage of hemolysis in presence of 1/8 MIC of metformin, NAC, and secnidazole were calculated as compared to the control. The OD values of the treated strains were expressed as a percentage of the OD values compared with the untreated control strain. The data shown are the means ± standard errors of three biological experiments with three technical replicates each. *, significant *P* < 0.05

Notably, metformin, NAC, and secnidazole-treated isolates exhibited a significant decrease in hemolysin activity compared to untreated isolates. The inhibition of hemolysin activity under the effect of metformin, NAC, and secnidazole was reduced by a percentage ranging between (10%-54%), (18%-80%), and (9%-35%) respectively at 1/8 MIC as shown in Fig. 5.

### Reduction of the tolerance against oxidative stress in *K. pneumoniae* tested isolates

**Fig. 6:**
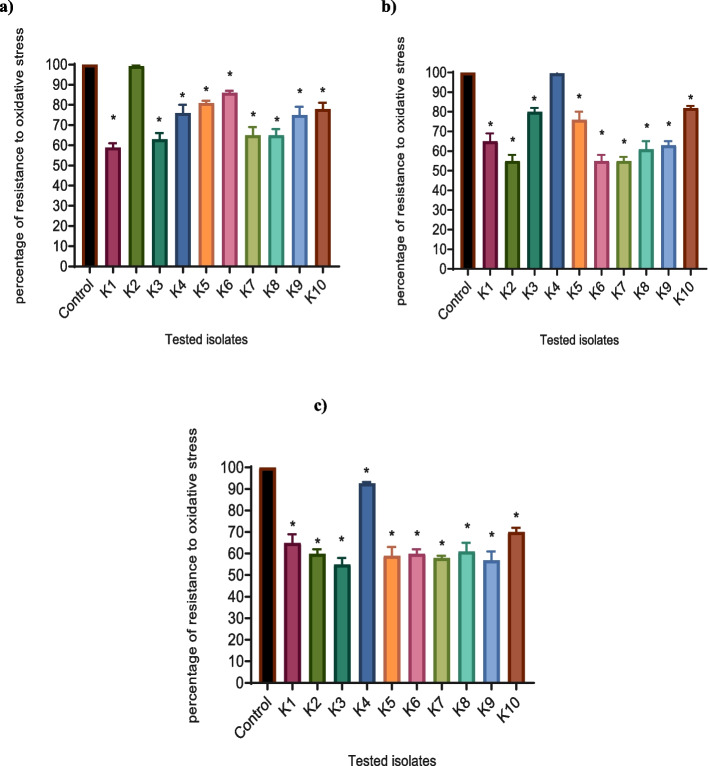
Inhibition of resistance to H_2_O_2_ in *K. pneumoniae* by **a**) metformin, **b**) NAC, and **c**) secnidazole. The modified disk test was carried out. A significant reduction of resistance to H_2_O_2_ was found with 1/8 MIC of metformin, NAC, and secnidazole-treated isolate as compared to control untreated bacteria. The results were expressed as a percentage of zone inhibition of the treated isolates compared with the untreated control strain. The data shown are the means ± standard errors of three biological experiments with three technical replicates each. *, significant *P* < 0.05

The impact of metformin, NAC, and secnidazole on the tolerance of *K. pneumoniae* to oxidative stress was investigated by testing the increasing hydrogen peroxide lethal effect on the growth of *K. pneumoniae* by the tested drugs. Except for K2 under the effect of metformin and for K4 under the effect of NAC, metformin, NAC, and secnidazole showed a significant impact in decreasing the tolerance of *K. pneumoniae* tested isolates to oxidative stress by a percentage of (14%-41%), (18% -45%), and (7%-45%), respectively (Fig. 6).

### Estimation of relative gene expression of virulence factors encoding genes in ***K. pneumoniae*** by qRT-PCR

For the genotypic experiment, the most resistant isolate (isolate 1) was selected as a representative example to confirm the inhibition ability of virulence factors of metformin, NAC, and secnidazole in *K. pneumoniae* at the molecular level, qRT-PCR was used to estimate the relative expression of the genes regulating the production of virulence factors in *K. pneumoniae* in treated and untreated isolate using qRT-PCR and was analyzed using the 2^−∆∆Ct^ method.

The relative expression levels of *rmpA, wcaG, fimH-1, mrkD, ureA,* and *khe* were significantly decreased in metformin, NAC, and secnidazole treated isolates in comparison to control untreated isolate. At sub-MIC, metformin was found to be more potent than NAC and secnidazole against *K. pneumoniae* virulence factors genes, decreasing expression levels of *rmpA, wcaG, fimH-1, mrkD, ureA,* and *khe* genes by 37.8% to 75%, in comparison to NAC (50–80%), and (52.5–87.5%), by secnidazole.

**Fig. 7:**
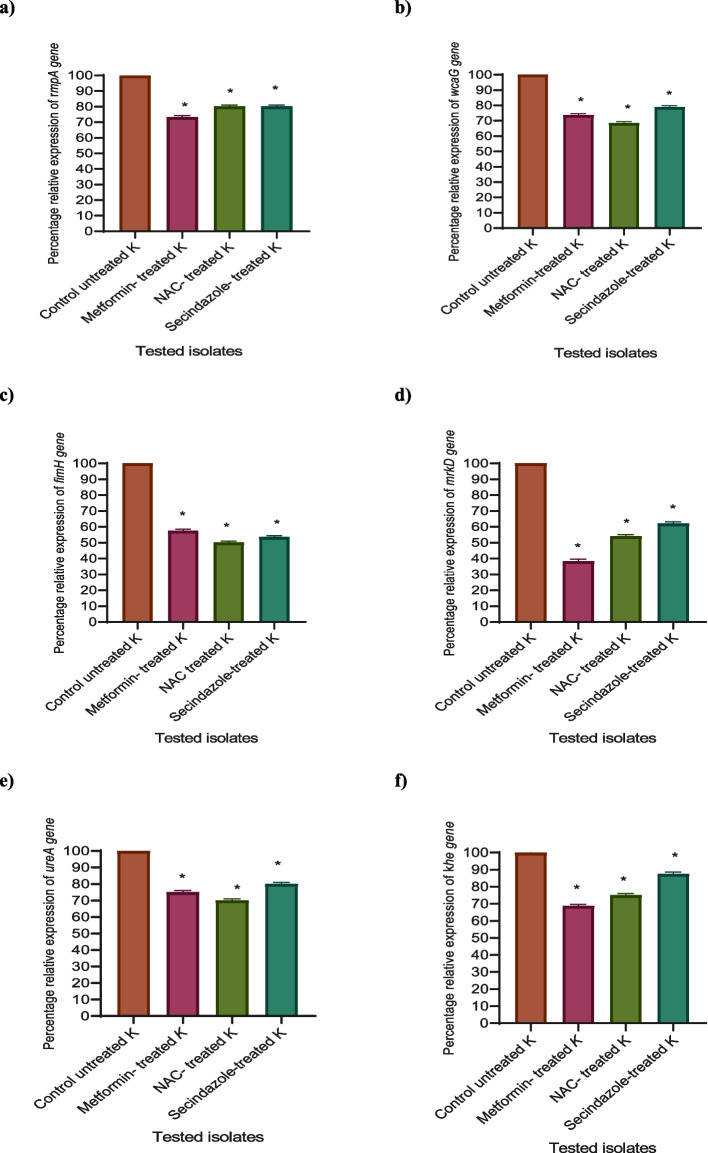
Down-regulation of virulence genes of *K. pneumoniae* by metformin, NAC, and secnidazole at 1/8 MIC produced a significant reduction in the expression levels of all tested virulence genes. **A**- *rmpA* gene, **B**- *wcaG* gene, **C**- *fimH-1*gene, **D**- *mrkD* gene, **E**- *ureA* gene, **F**- *khe* gene. The data shown represents the means ± standard errors. One WAY ANOVA test was used for statistical analysis. *, significant *P* < 0.05

Metformin decreased *rmpA* relative expression level by 26.67%, while NAC and secnidazole have a nearly identical effect on *rmpA* with a 20% inhibition effect. The *wcaG* gene was suppressed by 26.32%, 31.58%, and 21.05% in metformin, NAC, and secnidazole-treated isolates, respectively. Metformin, NAC, and secnidazole suppressed the *fimH-1* gene by 42.5%, 37.84%, and 47.5%, respectively. In addition, metformin inhibited the relative expression level of the *mrkD* gene by 62.16% and by 45.95% and 45.95% under the effect of secnidazole and NAC, respectively. Also, metformin, NAC, and secnidazole significantly lowered *ureA* relative expression levels by 25%, 30%, and 20%, respectively, while *khe* relative expression level was reduced by 31.25%% in metformin-treated isolate, 25% in NAC-treated isolate, and 12.5% in secnidazole-treated isolate (Fig. 7).

#### Mice survival assay

**Fig. 8:**
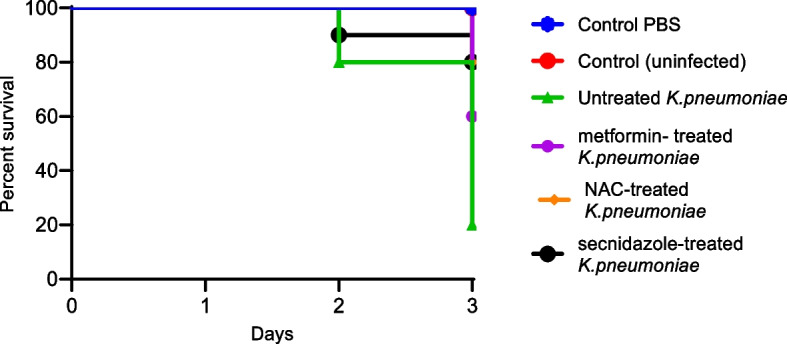
In vivo survival test of *K. pneumoniae*. Six groups of healthy mice, each consisting of 10 mice. We used two negative control groups, either injected with sterile PBS or left uninfected, and a positive control group injected with untreated *K. pneumoniae*. We injected metformin, NAC, and secnidazole-treated *K. pneumoniae* into three test groups. Mice were observed every day for three days using the Kaplan–Meier method, GraphPad Prism 8. All mice in the negative control groups survived, while only 20% of mice in the positive control group did. Metformin in sub-MIC protected 6 mice, conferring 60% protection. 6 mice survived. Meanwhile, NAC and secnidazole confer 80% protection from *K. pneumoniae*; 8 mice survived

The in vivo protective activities of Metformin, NAC, and secnidazole from *K. pneumoniae* pathogenesis was assessed using five groups of mice. The mice survival was reported for 3 successive days and plotted using the Kaplan–Meier method and significance (*P* < 0.05) was calculated using the Log-rank test, GraphPad Prism 8 (Fig. 8). All mice in negative control groups (un-inoculated or PBS injected) survived (100%). Only 20% of mice that were injected with untreated bacteria survived in the positive control group. As compared to mice inoculated with non-treated bacteria, mice treated with metformin in sub-MIC had a 60% increase in survival. Similarly, NAC and secnidazole showed a significant increase of 80% in survival rates (Fig. 8).

## Discussion

*K. pneumoniae* causes a wide variety of diseases in inpatients and is frequently responsible for antimicrobial-resistant opportunistic infections [38]. Newborns, the elderly, and immunocompromised individuals are more likely to contract *K. pneumoniae* infection [39]. Antibiotic resistance in bacteria is one of the most serious threats to public health and healthcare [40]. *K. pneumoniae* has been identified as a reservoir for antibiotic-resistant genes that can spread to other Gram-negative bacteria [41]. Currently, MDR *K. pneumoniae* infections have become a major problem due to a lack of effective antibiotics, which have resulted in an increased morbidity rate, longer hospitalizations, a higher mortality rate, and higher healthcare costs than infections caused by bacteria that are susceptible to antibiotics [42].

The need for novel antimicrobial therapy techniques to be developed, either alone or in combination with antibiotics, has become critical. In this context, "repurposing" (defined as searching for potential uses for existing drugs) has regained popularity. The use of these existing drugs in combination with antimicrobial treatments now in clinical trials is also being considered [14]. This concept focuses on designing or repurposing drugs that limit bacterial pathogenicity. Targeting bacterial virulence instead of their viability is a concept that has been reported in several publications [43]. As a result, anti-biofilm and anti-virulence techniques are becoming more acceptable as antimicrobial therapy [44].

Previous studies suggested that metformin, NAC, and secnidazole can be effective anti-virulence agents against some other pathogens and that metformin, NAC, and secnidazole are safe and effective in treating some health problems in neonates, we conducted a study in which metformin, NAC, and secnidazole were tested as anti-virulence agents against neonates isolates of MDR *K.pneumoniae*. Metformin has been shown to maintain maternal glycemic control in a previous study. Fetal and placental tissues contain clinically relevant levels of metformin (50%-100%) [45]. According to a meta-analysis study [46], metformin may have the same glycemic control as insulin and may be the most effective drug for preventing maternal and neonatal complications. In terms of NAC, A prior study also found that instilling NAC through a tracheal tube in already intubated babies is an effective, simple, and unique therapy approach for atelectasis [47]. Administration of NAC during pregnancy and after birth was safe, and can preserve cerebrovascular control, and boost levels of a neuroprotective protein that fights inflammation [48]. Moreover, Secnidazole is an analog to 5-nitroimidazoles group in which metronidazole is routinely used in conjunction with other antibiotics for the treatment of necrotizing enterocolitis [49].

In the current study, the antibacterial activity of the tested drugs metformin, NAC, and secnidazole was determined through MIC evaluation. Metformin and secnidazole were able to inhibit *K. pneumoniae* growth at 8 mg/ml, while N-acetylcysteine inhibited the growth at 16 mg/ml.

*K. pneumoniae* produces several virulence factors that facilitate their invasion of the host. *K. pneumoniae* capsule is a polysaccharide produced by all strains of *K. pneumoniae* that acts as a protective covering on the bacterium's surface, preventing phagocytosis, complement, and stimulation of the host inflammatory response [50]. In addition, *K. pneumoniae* can form a biofilm that protects it from both the host immune response and antibiotics. The creation of biofilm enables *K. pneumoniae* to stay for a long time on the epithelium and medical devices, making its eradication difficult [51]. Biofilms of *K. pneumoniae* may also contribute to gastrointestinal, respiratory, and urinary tract colonization, as well as the development of invasive infections, particularly in immunocompromised patients and infants [8, 52]. Importantly, *K. pneumoniae* produces urease enzyme as a virulence factor to utilize urea as a source of nitrogen for growth by its ability to hydrolyze it to ammonia and carbon dioxide [53]. The production of ammonia by urease can potentially be a major source of tissue damage and, in some cases, a critical role in pathogen persistence [54, 55]. On the other hand, bacterial proteases play vital functions in cell physiology, replication, and survival. Extracellular proteases are responsible for the destruction of host tissue as well as the degradation of host defense proteins including immunoglobulin A (IgA) [56]. Another type of enzyme is hemolysins are produced and promote erythrocyte lysis and it is common with the more severe forms of infection [50, 57].

Five virulence factors (biofilm formation, urease, proteases, hemolysins production, and resistance to oxidative stress) were assessed in *K. pneumoniae* in the presence and absence of 1/8 MIC (1 mg/ml, 2 mg/ml, and 1 mg/ml) for the tested potential inhibitors metformin, NAC, and secnidazole, respectively. Interestingly, the tested potential inhibitors had a significant inhibitory effect on the production of virulence factors.

By the current results, previous studies revealed that metformin significantly reduced the production of the virulence factors proteases and hemolysins in another Gram-negative bacteria *Pseudomonas aeruginosa* which shows a promising anti-virulence activity of metformin [58]. Moreover, metformin treatment of *Pseudomonas aeruginosa* markedly decreased biofilm formation, and increased the sensitivity to oxidative stress [59]. Also, metformin inhibits biofilm formation, proteases production, and hemolysins production in *Serratia marcescens* [60].

On the other hand, matching with the current study results, NAC was proved to have excellent effectiveness both in inhibiting biofilm formation and in destroying developed biofilms in *S. aureus*, *S. epidermidis*, *Escherichia coli*, *K. pneumoniae*, *Pseudomonas aeruginosa*, and *Proteus vulgaris* [61].In addition, Abdel-Bakry et al. (2017) reported that NAC has an inhibitory activity on the biofilm formation and urease production in *Proteus mirabilis* [62] which is in accordance with our results.

In addition similar to our findings, a previous study demonstrated that secnidazole reduced biofilm formation, and the production of proteases, and hemolysins in *Serratia marcescens* [23]. Also, Saleh et al. (2019) found that the influence of secnidazole on *Pseudomonas aeruginosa* proteases and hemolysins production was highly strong in inhibiting them [63].

Also, the anti-virulence activity of metformin, NAC, and secnidazole in the current study against biofilm formation, urease, proteases, hemolysins production, and tolerance to oxidative stress against *K. pneumoniae* is proportional to previous reports tested some potential anti-virulence agents. In a study, tea polyphenols at sub-MICs inhibited biofilm formation and protease production in *K. pneumoniae* [64]. In another study, curcumin possessed appropriate antibiofilm and anti-capsule activities of hypervirulent *K. pneumoniae* [65]. Also, Vitamin C inhibits biofilm and capsule formation in hypervirulent carbapenem-resistant* K. pneumoniae* [66]. Wang et al. 2021 studied ten flavonoid effects as antibiofilm agents and revealed that rutin is a potential one used in *K. pneumoniae* [67].

An important note needs to be highlighted that K2, and K4 isolates were not inhibited by metformin and NAC, respectively in testing the tolerance against oxidative stress. That may be due to intrinsic differences in their virulence factor production compared to the other tested strains. It is possible that these strains have evolved alternative mechanisms that are not affected by the tested drugs [68].

At the molecular level, *K. pneumoniae* virulence factors are encoded by certain virulence-regulating genes including *rmpA, wcaG, fimH-1, mrkD, ureA,* and *khe.* The *rmpA* and *wcaG* genes are responsible for *K. pneumoniae* capsule biosynthesis which boosts the ability of bacteria to evade phagocytosis by macrophages. On the other hand,* FimH* and *mrkD* genes encode fimbrial adhesins which mediate binding to the extracellular matrix to form the biofilm; while *ureA* regulates urease production and *khe* is responsible for hemolysins activity [10, 31, 69–71].

Considering the current data revealing that metformin, NAC, and secnidazole phenotypically decreased the production of virulence factors in *K. pneumoniae*, the relative expression level of *rmpA*, *wcaG*, *fimH*-1, *mrkD*, *ureA*, and *khe* genes regulating virulence factors production was assessed using qRT-PCR for more confirmation. Importantly, qRT-PCR revealed that metformin, NAC, and secnidazole significantly reduced the expression levels of all the tested genes that control the production of different virulence factors in *K. pneumoniae* which augment the phenotypic results. Similarly, the results of Namikawa et al. (2019) stated that the qRT-PCR results showed a significant downregulation by rifampicin for* rmpA* in hypervirulent *K. pneumoniae* [72]. Also, Vitamin C was reported as an efficient anti-virulence agent at the molecular level that suppresses the expression level of various virulence-associated genes including *rmpA* in *K. pneumoniae* [66]. The anti-virulence effects of metformin were attributable to its capacity to decrease expression of genes involved in QS [73].

It was crucial to characterize *K. pneumoniae* pathogenesis in vivo in light of recent findings that showed *K. pneumoniae* can produce fewer virulence factors when treated with metformin, NAC, and secnidazole. A mice survival in vivo model was used to evaluate the protective effects of the tested drugs in sub-MIC against *K. pneumoniae* pathogenesis. Metformin provided 60% protection to mice, while NAC and secnidazole provided 80% protection. Similar to our findings, a recent study reported that vitamin C was found to have a bactericidal effect against carbapenem-resistant hypervirulent *K. pneumoniae* in a mice infection model [66].

## Conclusion

According to our results, metformin, NAC, and secnidazole are effective inhibitors of virulence factors in *K. pneumoniae*. This suggests they can be used as adjuvant therapies as an alternative or in combination with traditional antibiotics in developing countries to combat *K. pneumoniae* infections, particularly neonatal sepsis. Such results can support the use of FDA-approved drugs as a new strategy to combat antimicrobial resistance.

## Data Availability

The authors confirm that the data supporting the findings of this study are available within the article.
